# Spontaneous pneumomediastinum; Time for a consensus

**DOI:** 10.34172/jcvtr.2022.26

**Published:** 2022-08-30

**Authors:** Santiago Campbell-Silva

**Affiliations:** Internal Medicine Unit, Mediláser Clinic, Florencia, Caquetá, Colombia

## Dear Editor,

 Considering the article published by Hülya Dirol and Hakan Keskin: Risk factors for mediastinitis and mortality in Pneumomediastinum,^1^ I would like to respectfully give my opinion on the so-called spontaneous pneumomediastinum.

**Figure 1:**
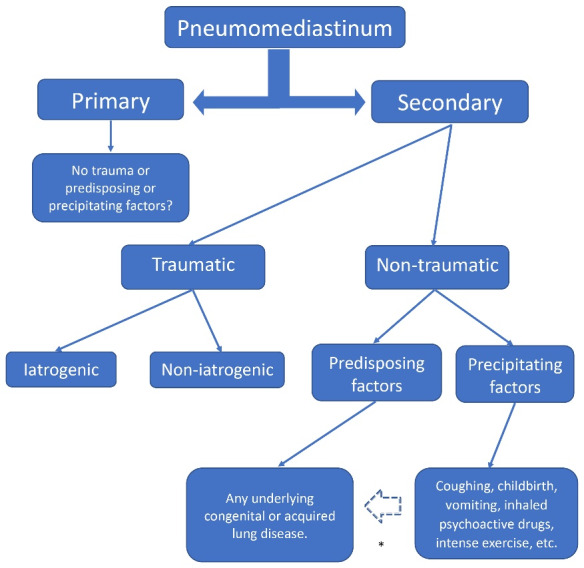
Cause of pneumomediastinum

 We agree in dividing pneumomediastinum into primary and secondary, but we consider that the primary is the truly spontaneous or idiopathic, terms that should be omitted and called primary. This pneumomediastinum cannot have any predisposing or precipitating factor. The secondary, can in turn be traumatic and non-traumatic, and the latter is where the predisposing and precipitating factors influence (Figure 1). According to current literature, any pneumomediastinum that is not traumatic is “spontaneous”, but it happens that the group called spontaneous is the one with the greatest triggering cause.

 This creates confusion and causes conceptual difficulty. We believe that the classification should evolve towards greater accuracy.

 For us, if a PM has a predisposing factor that compromises the pulmonary structure whether congenital, hereditary, or genetic (bronchiectasis, cystic fibrosis, surfactant alterations, etc.) or acquired (asthma, COPD, interstitial lung disease, COVID-19, etc.), it cannot be spontaneous because there is an underlying predisposing disease. Similarly, the pneumomediastinum that occurs due to a precipitating factor (coughing, labor, intense exercise, use of inhaled drugs, mechanical ventilation, etc.) in a healthy subject or with an underlying lung disease cannot be spontaneous either because there is an event immediate trigger that causes it.

 Truly primary pneumomediastinum is a rare event and very little reported in the medical literature.^2,3,4,5^

 I prefer a classification that considers not only the etiology of pneumomediastinum, but also helps to provide a correct diagnosis and is useful in guiding selective management strategies for better care, as well as being easy to remember and useful for teaching.

 In short, we 0believe that:

- The true definition and classification of pneumomediastinum are not in accordance with what is published in the current literature. The definition is misused.
- The term “spontaneous” pneumomediastinum should be omitted and called primary pneumomediastinum.
- Primary pneumomediastinum is that pneumomediastinum that occurs without any obvious causal factor (predisposing or precipitating), it must occur “spontaneously”.
- Secondary pneumomediastinum is that pneumomediastinum that occurs when there is an obvious causal factor (predisposing or precipitating or both).
- Primary pneumomediastinum under these conditions is an extremely rare condition.
- Secondary pneumomediastinum is the most frequently reported condition.
- Predisposing or precipitating factors can act alone or together; they are not exclusive.
